# Beyond Labels: Can Biomarkers and Treatable Traits Revolutionize Interstitial Lung Disease Care?

**DOI:** 10.3390/biomedicines13102467

**Published:** 2025-10-10

**Authors:** Francesco Amati, Anna Stainer, Stefano Aliberti

**Affiliations:** 1 Department of Biomedical Sciences, Humanitas University, Pieve Emanuele, 20072 Milan, Italy; 2 Respiratory Unit, IRCCS Humanitas Research Hospital, Rozzano, 20089 Milan, Italy

The classification of interstitial lung disease (ILD)s has traditionally relied on well-defined diagnostic labels, such as idiopathic pulmonary fibrosis (IPF), nonspecific interstitial pneumonia (NSIP), and hypersensitivity pneumonitis (HP) [1,2]. This classical approach is grounded in extensive clinical experience and research, providing a structured framework for understanding disease prognosis, guiding therapy selection, and facilitating communication among healthcare providers. Diagnostic labels easily offer established treatment protocols and clinical trials, enabling clinicians to deliver prognostic information with greater confidence. Despite its advantages, this system has notable limitations. ILDs are heterogeneous, and patients sharing the same diagnostic label may exhibit diverse symptoms, comorbidities, and treatment responses [3]. Some patients do not clearly fit into a single category, which can create uncertainty in management. Additionally, exclusive reliance on diagnostic labels may obscure modifiable factors that influence quality of life (QoL) or disease progression [4]. To address these challenges, an approach based on treatable traits (TT)s has emerged in ILD [5]. Rather than prioritizing the overarching disease label, this strategy identifies and targets specific, modifiable pulmonary, extrapulmonary, etiological, and lifestyle features in each patient, such as airway inflammation, pulmonary hypertension, or gastroesophageal reflux. Focusing on TTs enables clinicians to provide more personalized and adaptable care, adjusting interventions as the patient’s condition changes [5]. This approach is especially valuable for patients whose disease does not conform to classic patterns or who exhibit overlapping features from various ILDs [6]. It emphasizes the identification of modifiable clinical, biological, or molecular characteristics that inform therapeutic decisions beyond traditional diagnostic categories.

In ILD, biomarkers are crucial for identifying these TTs. Biomarkers are quantifiable biological indicators that reflect underlying pathophysiological processes or responses to therapy. In ILDs, biomarkers provide objective insights into disease mechanisms, addressing the limitations of clinical and radiological assessments, which often fail to capture the complexity and heterogeneity of ILD presentations and manifestations [7,8,9]. The identification and application of biomarkers offer promising avenues for early diagnosis, prognostic stratification, and the monitoring of treatment response [8]. For instance, active inflammation, progressive fibrosis, or immune dysfunction can be objectively assessed and monitored using specific biological markers. Serum biomarkers, including Serum Krebs von den Lungen-6 (KL-6), surfactant proteins A and D, and CC-chemokine ligand 18 (CCL18), have been linked to disease progression and prognosis [10,11,12].

Additionally, emerging multi-omic analysis techniques are enabling the identification of increasingly precise molecular profiles. Multi-omic analysis refers to the integrated study of various “omics” layers, including genomics (DNA), transcriptomics (RNA), proteomics (proteins), metabolomics (metabolites), and epigenomics (epigenetic modifications) [13]. By combining data from these different biological levels, a more comprehensive picture of ILD mechanisms, patient subtypes, and potential therapeutic targets might be identified [13]. Multi-omics approaches can reveal novel biomarkers for earlier and more accurate diagnosis, as well as for predicting disease progression and response to therapy. For instance, integrating genomic and transcriptomic data can help distinguish IPF from other forms of ILD, or even identify subgroups within a given diagnosis that have different prognoses or treatment needs [14,15]. Moreover, multi-omics analysis can uncover new disease pathways and mechanisms that are not apparent from clinical observation alone [16].

This deeper understanding can guide the development of targeted therapies, moving the field toward precision medicine [17]. Combinational biomarkers can optimize therapeutic resources by identifying patients who may benefit from specific antifibrotic or immunosuppressive therapies. Combinational biomarkers may also indicate suitability for novel pharmacological strategies under investigation. The identification of “high-risk patients” through the application of biomarkers can help minimize patients’ exposure to potentially harmful treatments. This could lead to better outcomes, improved patient adherence, and potentially decreased healthcare costs in the long term [18]. The accurate early prediction of disease behavior using biomarkers is particularly important for certain ILDs, such as sarcoidosis or connective tissue diseases (CTDs), where diagnosis often occurs in young adults and lifelong treatment is initiated based on clinical and radiological findings [19,20]. Establishing provisional biomarker thresholds can guide treatment decisions. These actionable thresholds facilitate the transition from theoretical biomarkers to practical tools that shape clinical management of ILD. For example, serum biomarker levels above a defined value may prompt more aggressive intervention in early disease stages, while lower levels may support treatment de-escalation to minimize side effects. A combination of biomarkers to identify those progressive pulmonary fibrosis (PPF) patients with a ‘high-risk signature’ could enrich clinical trial cohorts and avoid the need for antecedent progression when defining PPF for clinical trial enrolment [21]. This could lead to an enhanced trial efficiency as targeting patients more likely to benefit from therapy can increase statistical power and reduce sample size requirements, thereby speeding up the evaluation process and optimizing resource allocation [22,23]. From this perspective, dynamic biomarkers may serve as early surrogate endpoints, allowing for a faster assessment of efficacy by shortening endpoint durations [24]. As technologies advance and costs decrease, biomarker profiling is likely to become increasingly crucial in ILD, helping to unravel its complexity and improve patient outcomes.

Despite these advantages, implementing a TTs approach based on biomarkers in ILDs presents several challenges. The heterogeneity of ILDs and the complexity of their mechanisms hinder the definition of universal TTs. A multidisciplinary team (MDT) is essential, as interpreting and applying biomarker data requires the combined expertise of pulmonologists, radiologists, pathologists, and laboratory specialists [25]. Indeed, the majority of TTs observed within the ILD population in a recent study were found to be related to comorbidities and extra-respiratory manifestations, in which identifying reliable biomarkers may be even more complex [26]. This could lead to a risk of over-reliance on biomarkers that may not fully capture disease complexity or may be influenced by comorbidities, leading to inappropriate treatment choices. Furthermore, many proposed biomarkers lack robust validation across diverse ILD populations, raising concerns about generalizability [27]. Identifying clinically meaningful biomarkers and TTs requires robust longitudinal studies and the comprehensive integration of clinical, radiological, and molecular data. Moreover, biomarker assays can exhibit technical variability and may lead to the misclassification of ILD patients, thereby affecting trial outcomes and interpretation [28]. Implementing biomarker testing adds complexity, cost, and sometimes delays to trial enrollment; there may be disparities in access to testing [23,27]. Lastly, the regulatory landscape for biomarker-driven therapies remains complex, with uncertainties around standardization and reimbursement that could hinder widespread clinical adoption [28].

In conclusion, the integration of biomarkers into the clinical practice of ILDs, in synergy with an approach focused on TTs, represents a decisive turning point towards a more effective, personalized, and dynamic management of these complex conditions, with the ultimate goal of improving patients’ prognosis and QoL.

## Data Availability

Not applicable.
